# Functional *IGF1R* variant predicts breast cancer risk in women with preeclampsia in California Teachers Study

**DOI:** 10.1007/s10552-017-0942-7

**Published:** 2017-08-18

**Authors:** Mark J. Powell, Julie Von Behren, Susan Neuhausen, Peggy Reynolds, Christopher C. Benz

**Affiliations:** 1 Zero Breast Cancer, San Rafael, CA USA; 2 Cancer Prevention Institute of California, Berkeley, CA USA; 3 Beckman Research Institute of City of Hope, Duarte, CA USA; 4 Buck Institute for Research on Aging, Novato, CA USA

**Keywords:** Breast cancer risk, Preeclampsia, Insulin-like growth factor 1 receptor, Gestational hypertension, Age at first birth

## Abstract

**Purpose:**

Hypertension in pregnancy has been associated with decreased future risk of breast cancer in many but not all studies. In the Marin Women’s Study, pregnancy-induced hypertension was shown to interact with the T allele of a functional *IGF1R* gene variant, rs2016347, to result in lower breast density, as well as decreased breast cancer risk. Our objective was to explore these findings in a larger sample of women from the California Teachers Study (CTS).

**Methods:**

The CTS cohort consists of over 130,000 female educators. DNA was available from a nested case–control study, which included 2,030 non-Hispanic white women who developed breast cancer and 1,552 controls. The current study included all participants from the case–control group with a self-reported history of preeclampsia (80 cases/57 controls).

**Results:**

Comparing TT to GG genotypes revealed adjusted odds ratios of 0.38 (CI 0.13, 1.14) for all invasive breast cancers, 0.26 (CI 0.07, 0.89) for hormone receptor-positive (HR+) breast cancers, 0.15 (CI 0.04, 0.56) for those with age at first birth (AFB) < 30, and 0.10 (CI 0.02, 0.49) for those with AFB < 30 and HR+ breast cancers. Trend analysis yielded *p* values of 0.09, 0.03, 0.005, and 0.004 respectively, suggesting a biological effect for each T allele.

**Conclusion:**

Study findings indicate that the T allele of *IGF1R* variant rs2016347 is associated with a significant reduction in breast cancer risk in women with a history of preeclampsia, most marked for HR+ breast cancer and in women with AFB < 30.

## Introduction

Pregnancy is known to have a major impact on the developing breast and results in a transient increase, but a long-term decrease in the risk of developing breast cancer. Hypertensive disorders of pregnancy are characterized by the development of elevated blood pressure after the 20th week of pregnancy and affect 6–8% of all pregnancies. Many but not all epidemiologic studies have demonstrated a lower future risk of breast cancer in women reporting such a history, whether it be hypertension alone (gestational hypertension), or hypertension with proteinuria (preeclampsia). A pooled meta-analysis of 19 publications in 2013 reported a hazard ratio of 0.86 for preeclampsia and 0.83 for gestational hypertension [1]. Most of the larger cohort studies have reported similar reductions in risk [2–4]. Although not all published studies have demonstrated reduced risk, many were limited by small numbers or short follow-up period and involved women of varying ethnicities with presumed differences in the frequencies of inherited gene variants commonly known as single nucleotide polymorphisms (SNPs) [5].

Both gestational hypertension and preeclampsia are associated with inadequate invasion of the uterine vasculature by placental cytotrophoblasts, resulting in placental ischemia and the subsequent release of numerous biologically active factors such as cytokines, angiogenic factors, and growth factors/inhibitors that could impact the breast during this critical developmental period [6]. Many studies have reported lower serum IGF-1 (insulin-like growth factor 1) levels in women with preeclampsia as well as lower placental expression of IGF-1 [7–10]. Increased placental oxidation of the primary receptor for IGF-1, IGF1R (insulin-like growth factor 1 receptor), has also been demonstrated in these women [11].

IGF-1 is an important mediator of mammary terminal ductal formation during development, and increased activity of the gland’s IGF-1/IGF1R system has been shown to play a major promoting role in the development of breast cancer [12, 13]. A pooled data analysis of 17 studies demonstrated that women with higher circulating IGF-1 levels have a higher risk of breast cancer, and that this is especially true for ER+ (estrogen receptor-positive) breast cancer development [14]. The growth-promoting effects of IGF-1 are mediated primarily through IGF1R, and high cytoplasmic expression of IGF1R in terminal duct lobular units (TDLUs) has been associated with significantly increased risk of later-life breast cancer [15].

Prior work in the Marin Women’s Study (MWS) demonstrated that a history of hypertension in pregnancy is associated with lower later-life breast density and, furthermore, that this lower breast density is dependent on the inheritance of at least one T allele of rs2016347, a common and functional SNP located within the 3′UTR of *IGF1R* [16]. The MWS also demonstrated a statistically significant lower risk of breast cancer in women with a history of hypertension in pregnancy if they carry the TT genotype of rs2016347, although these findings were based on only 22 cancer cases [17]. The current study explored these findings in a larger sample of women from the California Teachers Study (CTS), in which an earlier analysis had failed to show any statistically significant breast cancer protective effect associated with a history of preeclampsia in either the most recent or any prior pregnancy [18].

## Methods

The California Teachers Study (CTS) is an ongoing prospective cohort study of breast cancer composed of 133,479 active and retired female public school teachers and administrators recruited in 1995 from the California State Teachers Retirement System. The baseline questionnaire (Q1) captured detailed information on height/weight, physical activity, menstrual/reproductive history, oral contraceptive use, menopausal hormone therapy use, and family history of cancer. A question about preeclampsia was asked on the second CTS questionnaire (Q2) in 1997. Cancer outcomes in the CTS are identified through annual linkage with the California Cancer Registry, a legally mandated statewide population-based cancer reporting system.

Blood samples were available from a case–control study established in 2012, which included 2,030 non-Hispanic white women who developed breast cancer after entry into the study and 1,552 controls without invasive or in situ breast cancer. The current study nests within this case–control study, and all participants with a self-reported history of preeclampsia were selected (80 cases/57 controls). Hazard ratios for breast cancer in women with preeclampsia were calculated using Cox proportional hazards models in SAS. Adjusted models included age at baseline, age at first birth, total number of births, body mass index (BMI) at baseline, age at menarche, and family history of breast cancer. Invasive breast cancer cases were annotated as hormone receptor positive (HR+) if they were either estrogen receptor positive (ER+) or progesterone receptor positive (PR+).

Genotyping was performed at the Beckman Research Institute of City of Hope using MGB TaqMan Probe Assays from Life Technologies. For these assays, the reaction mix in a final volume of 5 µl included 5–20 ng genomic DNA, 4.5 pmol of each primer, 1.25 pmol of each probe, and 1× TaqMan Genotyping Master Mix from Life Technology. PCR cycling included 40 cycles of a two-step PCR (95 °C for 15 s and 60 °C for 1 min) after an initial 10 min at 95 °C. PCR amplification and allelic discrimination were carried out on an ABI PRISM 7900 HT instrument. For genotyping the samples, 160× master PCR reaction mix for each SNP was prepared; after loading the Master Mix to the DNA samples, 9× of remaining Master Mix was loaded to non-template DNA wells for negative control. The call rate for rs2016347 was 97.1%, and the T allele frequency was 0.51 in all women tested in the CTS cohort.

## Results

Our study nested within an existing CTS case–control study and the distribution of participant covariates for the cases and controls are presented in Table 1. There were no statistically significant differences (Chi-square *p* value < 0.05) in covariates or covariate subgroups. The mean age at baseline was 55.4 in cases and 56.5 in controls.

**Table 1:** Comparison of characteristics of the study participants by breast cancer case status

	Total *n* = 137		Case status			
			Yes *n* = 80		No *n* = 57	
	*n*	%	*n*	%	*n*	%
Age at baseline (*p* = 0.63)						
<50	43	31	28	35	15	26
50–59	44	32	24	30	20	35
60–69	36	26	19	24	17	30
70+	14	10	9	11	5	9
Age at first live birth (*p* = 0.21)						
<25	58	42	29	36	29	51
25–29	47	34	31	39	16	28
30+	30	22	19	24	11	19
Family history breast cancer (*p* = 0.59)						
No	110	80	63	79	47	82
Yes	27	20	17	21	10	18
Body mass index (*p* = 0.59)						
<25	55	40	32	40	23	40
25.0-29.9	40	29	26	33	14	25
30+ obese	36	26	18	23	18	32
Age at menarche (*p* = 0.55)						
≤11 years	37	27	18	23	19	33
12–13 years	77	56	47	59	30	53
≥14 years	20	15	13	16	7	12
Total number of live births (*p* = 0.77)						
1	22	16	14	18	8	14
2	56	41	35	44	21	37
3	38	28	21	26	17	30
4+	19	14	9	11	10	18

When comparing women with preeclampsia who have the TT genotype to those with the GG genotype, which would be expected to demonstrate the maximum effect of the T allele, the adjusted odds ratio for invasive breast cancer was 0.38 (CI 0.13, 1.14), *p* = 0.14, and thus did not reach statistical significance (see Table 2). When looking at those 61 cases known to be HR+, which includes ER+ or PR+ cases, the adjusted odds ratio was 0.26 (CI 0.07, 0.89), *p* = 0.03.

**Table 2:** Adjusted odds ratios for invasive breast cancer in non-Hispanic white women with history of preeclampsia for *IGF1R* rs2016347 TT versus GG genotype

Rs2016347 genotype	All cases with breast cancer (*n* = 137)	HR + cases only (*n* = 118)	AFB < 30 All cases (*n* = 106)	AFB < 30 HR + cases only (*n* = 92)
TT versus GG	OR 0.38 (CI 0.13, 1.14) *p* = 0.14	OR 0.26 (CI 0.07, 0.89) *p* = 0.033	OR 0.15 (CI 0.04, 0.56) *p* = 0.013	OR 0.10 (CI 0.02, 0.49) *p* = 0.008

Adjusted for age, parity, age first live birth (except in age first birth stratifications), first-degree relative with breast cancer, age menarche, and BMI.

Models were stratified by age at first birth (AFB) under or over age 30 and by premenopausal or postmenopausal case status to assess possible variations in the associations observed. Among women with an AFB under 30, those with the TT genotype when compared to the GG genotype demonstrated an adjusted odds ratio of 0.15 (CI 0.04, 0.56), *p* = 0.01, when looking at all types of invasive breast cancer. When looking at just HR+ breast cancer in women with AFB under 30, the adjusted odds ratio was 0.10 (CI 0.02, 0.49), *p* = 0.008. Stratification by pre- and postmenopausal breast cancer case status failed to demonstrate any statistically significant findings (data not shown).

In an effort to determine the biologic importance of just one T allele, the GT as well as the TT genotype was compared to the GG genotype, and the results of this analysis are provided in Table 3. In the overall as well as the stratified results, having one T allele has an intermediate effect; however, this was not statistically significant in the overall group with trend analysis demonstrating a *p* value of 0.09. Trend analysis was significant in the HR+ only group, *p* = 0.03, the AFB under 30 group, *p* = 0.005, and the group containing both HR+ cases only and AFB under 30, *p* = 0.004. This suggests that one T allele likely exerts a biologic effect.

**Table 3:** Adjusted odds ratios for invasive breast cancer in non-Hispanic white women with history of preeclampsia for one and two T alleles compared to reference *IGF1R* rs2016347 GG genotype

Rs2016347 genotype	All cases with breast cancer (*n* = 137)	HR + cases only (*n* = 118)	AFB < 30 All cases (*n* = 106)	AFB < 30 HR + cases only (*n* = 92)
GT versus GG	OR 0.53 (CI 0.19, 1.46) *p* = 0.23	OR 0.57 (CI 0.19, 1.74) *p* = 0.32	OR 0.34 (CI 0.12, 1.12) *p* = 0.15	OR 0.30 (CI 0.06, 1.17) *p* = 0.12
TT versus GG	OR 0.38 (CI 0.13, 1.14) *p* = 0.14	OR 0.26 (CI 0.07, 0.89) *p* = 0.033	OR 0.15 (CI 0.04, 0.56) *p* = 0.013	OR 0.10 (CI 0.02, 0.49) *p* = 0.008
Trend analysis	*p* = 0..09	*p* = 0.030	*p* = 0.005	*p* = 0.004

Adjusted for age, parity, age first live birth (except in age first birth stratifications), first-degree relative with breast cancer, age menarche, and BMI.

We also examined the risk for breast cancer in women with preeclampsia in the full CTS cohort without regard to rs2016347 genotype, providing a 7-year update to the earlier CTS findings and utilizing preeclampsia in any pregnancy as the exposure of interest [18]. Presently, there are 58,043 parous, non-Hispanic white women with known history of preeclampsia status, which includes 3,006 invasive breast cancer cases. In this updated analysis, the adjusted hazard ratio for invasive cancer in non-Hispanic white women with a history of preeclampsia in any pregnancy is 0.94 (CI 0.81, 1.08) after adjustment for age, age at first birth, total number births, BMI at baseline, age of menarche, and history of first-degree relatives with breast cancer.

## Discussion

The CTS results suggest significant breast cancer protection in women with preeclampsia who possess the *IGF1R* rs2016347 TT genotype, specifically in those with age at first birth under 30 and for subsequent development of HR+ breast cancer. These results are consistent with prior MWS findings, and both studies demonstrate that a lower risk of breast cancer in women with a hypertensive disorder of pregnancy is dependent on carrying the same functional rs2016347 variant. The protective TT genotype of rs2016347 is presumed to be functional both by its clinical impact (discussed below) and by its association with a reduction in IGF1R mRNA expression in multiple normal human tissues [19].

Our findings suggest a SNP-exposure interaction, as the rs2016347 SNP alone has not been shown in multiple genome-wide studies to be associated with changes in the risk of either breast cancer or preeclampsia. We speculate that this functional SNP genotype may interact with one or more of the numerous alterations in hormones and growth factors that occur as a result of the placental insufficiency seen in women with hypertensive disorders of pregnancy. Documented changes in these women include decreased estrogen levels and lower IGF-1 levels with higher levels of IGF-1 binding proteins [20].

Early full-term pregnancy has long been known to have a protective effect on future breast cancer risk. Animal models have demonstrated that presumed epigenetic changes occur in rat mammary glands that persist long after pregnancy and strongly protect against mammary tumorigenesis [21]. Humans also have a lasting genomic signature that results from pregnancy that may explain its long-term preventive effect [22]. Identified changes include higher chromatin condensation and increased histone methylation [23]. Our findings support the possibility of a permanent risk-reducing change induced in the breast tissue of women who experience a hypertensive disorder of pregnancy and who carry the rs2106347 T allele(s). Indeed, Katz et al. demonstrated in mice that parity results in significant and persistent hypermethylation of *IGF1R* and, furthermore, that this hypermethylation was associated with a reduction in mammary gland IGF1R mRNA expression [24]. Carrying the risk-reducing rs2106347 T allele(s) may further reduce mammary gland IGF1R mRNA expression by yet another epigenetic mechanism. MicroRNAs are small (~22 nucleotides) non-coding transcripts known to epigenetically reduce the expression of target transcripts at the translational level. By computational analysis, the T allele of rs2016347 appears to create a high-affinity binding site for miR-432 within the *IGF1R* 3′UTR, providing a putative mechanism for the predicted functionality of rs2016347 and the T allele dosage effect observed in both our CTS analysis and in the GTEx dataset [19].

Animal models have also demonstrated that IGF-1 can obliterate the pregnancy-associated protection against breast cancer by increasing ERα activation [25]. With regard to this pro-tumorigenic mammary gland interaction between IGF1R and ERα activation, our CTS findings with respect to HR+ breast cancer development are consistent with those of Winder et al. who found better outcomes for women with ER+ breast cancers treated with the antiestrogen tamoxifen who carry the T allele of rs2016347 [26].

A potential early application of these findings could lie in risk prediction. Although efforts at adding SNPs to risk assessment models have resulted in only an incremental improvement in risk prediction across the general population, there may be subgroups of women such as those identified here who may benefit significantly from the addition of a specific SNP to their individualized risk assessment [27]. According to the US Census, there are about 90 million parous women in the US, and close to 9 million of these women are expected to have a history of pregnancy-induced hypertension in at least one of their pregnancies. These women, with rs2016347 genotyping, might benefit from a more personalized estimate of their future breast cancer risk.

This study possesses a number of significant strengths. First and foremost, it presents data which are consistent with a previous study, thus the findings have now been demonstrated in two analyses performed at different times by separate research teams utilizing distinct cohorts, both finding statistically significant breast cancer protection in women with a history of hypertension in pregnancy who carry the rs2016347 TT genotype. Both analyses have excellent data on reproductive history and other breast cancer risk factors. Although the genotyping process was completed at different labs, the same assay approach and reagents were utilized in both studies, with resultant high call rates.

One potential limitation of our comparison between this study and the MWS study is that the “definition” of hypertension in pregnancy differed in the two studies. In the MWS, participants were asked if they had hypertension in pregnancy, while in the CTS participants were asked if they had preeclampsia in pregnancy. Thus, the CTS would not include women with gestational hypertension. However, these disorders are both associated with similar decreases in future breast cancer risk as shown in the referenced meta-analysis [1]. Moreover, the Child Health & Development Studies reported that women with recorded increases in blood pressure during pregnancy, even without reaching the clinical threshold of hypertension, also experience a significant decrease in future breast cancer risk [28, 29]. This suggests that the findings of this study could impact an even larger number of women including those who have not received a diagnosis of gestational hypertension or preeclampsia. Another potential study limitation is that the CTS used self-reported data for their determination of preeclampsia. Although this may introduce recall bias, a systematic review of maternal recall of all hypertensive disorders in pregnancy found a specificity of >90%, indicating that misclassification was probably not a significant factor in this CTS analysis [30].

In addition, the current study included only non-Hispanic white women. This was designed to make our CTS analysis more comparable to that of the MWS, but clearly limits generalizability. The small numbers resulting from the relatively low incidence rates of both preeclampsia and breast cancer may have precluded demonstrating statistical significance of relatively low effect size, as evidenced by the lack of statistical significance when looking at all cases of invasive breast cancer by genotype despite an adjusted OR of 0.38. Thus, our CTS findings warrant replication and further study in other large cohorts with similar access to both pregnancy hypertension history and later-life breast cancer risk.

## Conclusion

The results provided from this CTS analysis suggest significant breast cancer protection in women with preeclampsia who inherit the protective TT genotype for the *IGF1R* SNP rs2016347. Stratification of participants demonstrated that this protection is increased in women with age at first birth under 30, and for the later-life development of HR+ breast cancer. The rs2016347 T allele is common in many ethnicities and is likely functional, as recent studies have associated this variant with lower normal tissue expression of IGF1R mRNA and better treatment responses and patient survival from HR+ breast cancer.

The protective T allele appears to encode a new microRNA (miR-432) binding site within the *IGF1R* 3′UTR, offering a potential mechanistic hypothesis for the functionality of rs2016347 and its ability to interact with alterations of hormones and/or growth factors characteristic of hypertensive disorders of pregnancy and, thereby, to imprint the immature gland with a lasting protective effect from later-life tumorigenesis. Future proof of the mechanistic process may not only help explain why certain pregnancy events can protect some but not all women from breast cancer, but could also foster the design of much needed new breast cancer prevention strategies.

## Abbreviations

IGF1R: Insulin-like growth factor 1 receptor
SNP: Single nucleotide polymorphism
BMI: Body mass index
MWS: Marin Women’s Study
CTS: California Teachers Study
IGF-1: Insulin-like growth factor 1
3′UTR: Three prime untranslated region
MiRNA: MicroRNA
AFB: Age at first birth
HR+: Hormone receptor positive
ER+: Estrogen receptor positive
TDLU: Terminal duct lobular unit

## References

[1] Kim JS, Kang EJ, Woo OH, Park KH, Woo SU, Yang DS (2013). The relationship between preeclampsia, pregnancy-induced hypertension and maternal risk of breast cancer: a meta-analysis. Acta Oncol.

[2] Opdahl S, Romundstad PR, Alsaker MD, Vatten LJ (2012). Hypertensive diseases in pregnancy and breast cancer risk. Br J Cancer.

[3] Terry MB, Perrin M, Salafia CM, Zhang FF, Neugut AI, Teitelbaum SL (2007). Preeclampsia, pregnancy-related hypertension, and breast cancer risk. Am J Epidemiol.

[4] Innes KE, Byers TE (2004). First pregnancy characteristics and subsequent breast cancer risk among young women. Int J Cancer.

[5] Calderon-Margalit R, Friedlander Y, Yanetz R, Deutsch L, Perrin MC, Kleinhaus K (2009). Preeclampsia and subsequent risk of cancer: update from the Jerusalem Perinatal Study. Am J Obstet Gynecol.

[6] Reslan OM, Khalil RA (2010). Molecular and vascular targets in the pathogenesis and management of the hypertension associated with preeclampsia. Cardiovasc Hematol Agents Med Chem.

[7] Peng HY, Xue M, Xia AB (2011). Study on changes of IGF-I and leptin levels in serum and placental tissue of preeclampsia patients and their associativity. Xi Bao Yu Fen Zi Mian Yi Xue Za Zhi..

[8] Kharb S, Panjeta P, Ghalaut VS, Bala J, Nanda S (2016). Biomarkers in preeclamptic women with normoglycemia and hyperglycemia. Curr Hypertens Rev.

[9] Dubova EA, Pavlov KA, Lyapin VM, Kulikova GV, Shchyogolev AI, Sukhikh GT (2014). Expression of insulin-like growth factors in the placenta in preeclampsia. Bull Exp Biol Med.

[10] Kharb S, Nanda S (2017). patterns of biomarkers in cord blood during pregnancy and preeclampsia. Curr Hypertens Rev.

[11] Robajac D, Masnikosa R, Miković Ž, Mandić V, Nedić O (2015). Oxidation of placental insulin and insulin-like growth factor receptors in mothers with diabetes mellitus or preeclampsia complicated with intrauterine growth restriction. Free Radic Res.

[12] Ruan W, Kleinberg DL (1999). Insulin-like growth factor I is essential for terminal end bud formation and ductal morphogenesis during mammary development. Endocrinology.

[13] Christopoulos PF, Msaouel P, Koutsilieris M (2015). The role of the insulin-like growth factor-1 system in breast cancer. Mol Cancer.

[14] Key TJ, Appleby PN, Reeves GK, Roddam AW, Endogenous Hormones and Breast Cancer Collaborative Group (2010). Insulin-like growth factor 1 (IGF1), IGF binding protein 3 (IGFBP3), and breast cancer risk: pooled individual data analysis of 17 prospective studies. Lancet Oncol.

[15] Tamimi RM, Colditz GA, Wang Y, Collins LC, Hu R, Rosner B (2011). Expression of IGF1R in normal breast tissue and subsequent risk of breast cancer. Breast Cancer Res Treat.

[16] Prebil LA, Ereman RR, Powell MJ, Jamshidian F, Kerlikowske K, Shepherd JA (2014). First pregnancy events and future breast density: modification by age at first pregnancy and specific VEGF and IGF1R gene variants. Cancer Causes Control.

[17] Powell M, Prebil LA, Rose S, Jamshidian F, Benz C, Ereman R. Insulin-like growth factor-1 receptor variant associated with decreased breast cancer risk in women with pregnancy-induced hypertension. San Antonio Breast Cancer Symposium [abstract P3-07-03] 2013

[18] Ma H, Henderson KD, Sullivan-Halley J, Duan L, Marshall SF, Ursin G (2010). Pregnancy-related factors and the risk of breast carcinoma in situ and invasive breast cancer among postmenopausal women in the California Teachers Study cohort. Breast Cancer Res.

[19] National Human Genome Research Institute, Genotype-Tissue Expression Project. GTEx Portal Version V6p, Accessed 10 January 2017

[20] Altinkaynak K, Aksoy HH, Bakan E, Kumtepe Y (2003). Serum IGF-I and IGFBP-3 in healthy pregnancies and patients with preeclampsia. Clin Biochem.

[21] Blakely CM, Stoddard AJ, Belka GK, Dugan KD, Notarfrancesco KL, Moody SE (2006). Hormone-induced protection against mammary tumorigenesis is conserved in multiple rat strains and identifies a core gene expression signature induced by pregnancy. Cancer Res.

[22] Barton M, Santucci-Pereira J, Russo J (2014). Molecular pathways involved in pregnancy-induced prevention against breast cancer. Front Endocrinol (Lausanne).

[23] Cao R, Wang L, Wang H, Xia L, Erdjument-Bromage H, Tempst P (2002). Role of histone H3 lysine 27 methylation in Polycomb-group silencing. Science.

[24] Katz TA, Liao SG, Palmieri VJ, Dearth RK, Pathiraja TN, Huo Z (2015). Targeted DNA Methylation Screen in the Mouse Mammary Genome Reveals a Parity-Induced Hypermethylation of IGF1R That Persists Long after Parturition. Cancer Prev Res (Phila).

[25] Thordarson G, Slusher N, Leong H, Ochoa D, Rajkumar L, Guzman R (2004). Insulin-like growth factor (IGF)-I obliterates the pregnancy-associated protection against mammary carcinogenesis in rats: evidence that IGF-I enhances cancer progression through estrogen receptor-alpha activation via the mitogen-activated protein kinase pathway. Breast Cancer Res.

[26] Winder T, Giamas G, Wilson PM, Zhang W, Yang D, Bohanes P (2014). Insulin-like growth factor receptor polymorphism defines clinical outcome in estrogen receptor-positive breast cancer patients treated with tamoxifen. Pharmacogenomics J.

[27] Chlebowski RT (2017). Improving breast cancer risk assessment versus implementing breast cancer prevention. J Clin Oncol.

[28] Cohn BA, Cirillo PM, Christianson RE, van den Berg BJ, Siiteri PK (2001). Placental characteristics and reduced risk of maternal breast cancer. J Natl Cancer Inst.

[29] Cirillo PM, Benz CC, Cohn BA (2016). Comment on: ‘Hypertensive diseases in pregnancy and breast cancer risk’. Br J Cancer.

[30] Stuart JJ, Bairey Merz CN, Berga SL, Miller VM, Ouyang P, Shufelt CL (2013). Maternal recall of hypertensive disorders in pregnancy: a systematic review. J Womens Health (Larchmt).

